# A thematic analysis of tobacco industry responses to the Scottish Government’s consultation on e-cigarette regulation

**DOI:** 10.1093/heapro/daae145

**Published:** 2024-11-21

**Authors:** Billie Hamilton, Ruth Ponsford, Greg Hartwell

**Affiliations:** London School of Hygiene and Tropical Medicine, 15-17 Tavistock Place, London WC1H 9SH, UK; London School of Hygiene and Tropical Medicine, 15-17 Tavistock Place, London WC1H 9SH, UK; London School of Hygiene and Tropical Medicine, 15-17 Tavistock Place, London WC1H 9SH, UK

**Keywords:** vaping, e-cigarettes, tobacco industry, government consultations

## Abstract

E-cigarette use is increasing rapidly across the world. Governments have begun to tighten regulation principally to prevent uptake by young people and non-smokers. As tobacco industry ownership of the e-cigarette market grows, it is important to be aware of how industry is seeking to influence the regulation of e-cigarettes. Using thematic analysis, this research examines the explicit arguments and implicit tactics used in responses from the tobacco industry and linked organizations to Scotland’s 2022 consultation ‘Vaping Products—Tightening Rules on Advertising and Promoting’. The themes that emerged in the analysis were compared to tactics and arguments identified in past research on the tobacco industry to look for continuations and divergences. While the research finds continuation of historic arguments and tactics being used in the submissions, it also highlights important novel tactics and framings employed by tobacco industry actors, including incorporating outdated data and calling for UK-wide policy despite this being a devolved issue. Policymakers must remain alert to the strategies being used by the tobacco industry, so they are able to prioritize public health rather than the interests of industries that put profit before health.

**CONTRIBUTION TO HEALTH PROMOTION**

- This thematic analysis highlights the tactics used by the tobacco industry and linked organizations to attempt to influence e-cigarette policy in Scotland.
- It is important that policymakers and the public are aware of these strategies, so that they can restrict industry’s undue influence over population health.
- Improved awareness of this can help strengthen health promotion by countering industry’s attempts to shape decisions and policy related to our health.

## INTRODUCTION

### The rise in use of e-cigarettes

The number of people vaping has risen rapidly over the past decade; 2023 estimates show 82 million people using e-cigarettes worldwide, up from 68 million in 2020 (Jerzyński and Stimson, 2023). In comparison to other countries that have taken a far more regulatory or cautious approach to the emergence of e-cigarettes, UK governments have, up to recently, had a more relaxed approach, allowing e-cigarettes to be available on the consumer market rather than being banned or available only as a prescription product. Central government has also encouraged their use among smokers as a cessation aid, and the independent review by Khan ‘Making Smoking Obsolete’ (Khan, 2022), commissioned by the government, promotes e-cigarettes as a substitute for smoking. Outside Europe, many countries have banned the general use of e-cigarettes—Australia, for instance, has adopted a prescription-only model.

In the UK, the latest 2023 Action on Smoking and Health (ASH) figures show that there are 4.7 million adult users, up from 0.8 million in 2012 (ASH, 2023a). This makes up 8.3% of the UK population, the majority of whom are current or ex-smokers (ASH, 2023a).

Concerningly, e-cigarette use is also increasing among young people; in the UK, 7.6% of young people (aged 11–17) are current e-cigarette users, rising to 15% when accounting only for 16- to 17-year olds (ASH, 2023b). The number of young people regularly using e-cigarettes in 2023 has tripled since 2021 (ASH, 2023b), prompting widespread media and political interest.

### Safety of e-cigarettes and their effectiveness for smoking cessation

There have been a large number of reviews of the safety of e-cigarettes as well as their effectiveness as smoking cessation tools. A 2015 Public Health England’s (PHE) study concluded that e-cigarettes were around 95% less harmful than cigarettes (PHE, 2015). However, a recent report from the Office for Health Improvement and Disparities (OHID) found that the use of e-cigarettes is not risk-free, and their long-term effects remain unknown (OHID, 2022). A 2023 systematic review on health outcomes of e-cigarette use meanwhile found conclusive evidence that e-cigarettes could lead to poisoning and immediate inhalation toxicity, particularly in children and young people, and addiction in non-smokers (Banks *et al*., 2023), though some of these outcomes are likely to be associated with the use of unregulated e-cigarettes. The study also ‘found strong evidence that young never-smokers and non-smokers who use e-cigarettes are about three times as likely as non-users to start smoking tobacco and to become regular smokers’ (Banks *et al*., 2023). A systematic review and meta-analysis of e-cigarette use and future smoking initiation found that while there was a positive association between the two, the analysis could not discern whether this was causal (Chan *et al*., 2021). In terms of effectiveness, a 2022 Cochrane Review ‘Electronic cigarettes for smoking cessation’ found high certainty evidence that people randomized to e-cigarettes had higher quitting rates than those assigned to nicotine replacement therapy (NRT) (Hartmann-Boyce *et al*., 2022). However, of the 78 studies included, only 10 were deemed to be at low risk of bias (Hartmann-Boyce *et al*., 2022).

Industry-funded research remains an issue for the evidence base; a recent study found that the odds of finding no harm related to e-cigarettes were 67 times higher if the research was funded by the tobacco industry than if it was non-industry funded (Pisinger *et al*., 2019). Furthermore, the 95% less harmful figure has continued to be used in annual evidence updates on e-cigarettes by OHID despite it being controversial, with some questioning the methodology of the study it derived from, including its links to the tobacco industry (McKee and Capewell, 2015).

### The role of the tobacco industry

As the University of Bath, UK, has demonstrated, the tobacco industry has ‘invested in, developed and marketed various newer nicotine and tobacco products’ (Tobacco Tactics, 2023a) in a bid to tackle their shrinking profits as increasing tobacco control measures are implemented. The tobacco industry’s stake in the e-cigarette market grew rapidly from 20% in 2014 to 44% in 2019 (Tobacco Tactics, 2023a). Marketing tactics, product formulation and ease of access to e-cigarettes have been highlighted as reasons for their popularity in the younger generation in particular (Berg, 2015; Cancer Research UK, 2021; Gendall and Hoek, 2021; Notley *et al*., 2021; ASH, 2023a; Smith and Hilton, 2023; Tobacco Tactics, 2023a). While many are calling for tighter regulations of e-cigarettes, the tobacco industry refutes this, claiming that these products are in fact a harm reduction tool that can save lives.

The tobacco industry has a long history of interference in regulation of their products which has been uncovered most comprehensively through the publication of the Truth Tobacco Industry Documents (Truth Tobacco Industry Documents, no date, ‘Industry Documents Library’), an archive of 14 million tobacco industry documents detailing their strategies on advertising, scientific research and political activity. This led to the development of the world’s first public health treaty—The World Health Organization (WHO) Framework Convention on Tobacco Control (FCTC) ratified by 181 countries. Article 5.3 requires countries to protect their policymaking from the vested interests of the tobacco industry (WHO, 2003).

Yet despite the implementation of this treaty, there is evidence of the industry’s continued attempts to influence policies that will affect their profit margins including ‘on-off claims of commitment to “harm reduction” each time the industry is seriously threatened’ (Gilmore and Dance, 2023, p. 99). Gilmore and Dance’s study (Gilmore and Dance, 2023, p. 99) on the history of tobacco control finds that ‘there is little that major tobacco companies will not do to expand sales and maximize profits’. Government consultations are a key area where industry seeks to protect and expand their profits.

Despite Article 5.3 in the FCTC, tobacco companies have to date been able to respond to government consultations on product regulation which has become a key component of their lobbying work. Previous research on tobacco industry responses to government consultations (Hatchard *et al.*, 2014; Ulucanlar *et al.*, 2014; Evans-Reeves *et al*., 2015; Lie *et al*., 2018; Ikegwuonu *et al*., 2022) has identified common tactics used to contest policy changes that may undermine their economic interests. This was evident in responses to the UK’s consultation on standardized packaging (SP) for cigarettes, for instance (Hatchard *et al.*, 2014; Ulucanlar *et al.*, 2014; Evans-Reeves *et al*., 2015; Lie *et al*., 2018). Tactics identified included use of low-quality and industry-funded evidence in submissions, creating high administrative burdens for government through extremely lengthy submissions and commissioning new evidence (Hatchard *et al.*, 2014; Ulucanlar *et al.*, 2014; Evans-Reeves *et al*., 2015; Lie *et al*., 2018).

### Scottish consultation on vaping products

The Scottish Government’s 2013 Tobacco Control Strategy aims to make Scotland tobacco-free by 2034. As health and social care is a devolved power, the Scottish Government have led the approach to e-cigarette legislation within Scotland. In 2015, the Scottish Government (Scottish Government, 2015) commissioned their first consultation on e-cigarettes to obtain views on measures ‘to regulate the sale and use of electronic cigarettes and to strengthen tobacco control in Scotland’. The 2015 consultation received 172 responses including 11 responses from e-cigarette or tobacco industry actors. Eighty-eight per cent of consultation responses supported the introduction of legislation to ban the sale of e-cigarettes and liquids to those under 18. This came into force in the Health (Tobacco, Nicotine etc. and Care) (Scotland) Act 2016.

In 2022, due to growing concerns about the number of under-18s and non-smokers using e-cigarettes and the unknown harms associated with long-term use, a subsequent consultation (Scottish Government, 2022a) was commissioned to obtain views on the tightening of rules on advertising vaping products. Restricting the visibility, promotion and advertising of e-cigarettes, and instead positioning them as stop-smoking aids, has been the Scottish Government’s preference while the evidence base is being developed. Their proposed additional regulations (Scottish Government, 2022b) included advertising restrictions and ending brand-sharing, free distribution, nominal pricing, and sponsorship of activities, events or people in Scotland.

### What this research seeks to add

Globally, governments are wrestling with the issue of e-cigarette regulation, wanting to reduce its increasing appeal to children and young people while some are also seeking to preserve it as a cessation tool for current and ex-smokers. As outlined above, tobacco industry expansion into the e-cigarette market is still a relatively new phenomenon, and research into how they are attempting to influence e-cigarette policy is only recently starting to emerge (Patanavanich and Glantz, 2021; Ikegwuonu *et al*., 2022).

It is essential for public health researchers and policymakers to understand the role that actors, including the tobacco industry, have in shaping health and policy. The tobacco industry’s profit-making motivations conflict with health promotion, and so policymakers must be made aware of the tactics used by the industry so they can protect policy from undue influence and conflicts of interest.

Building upon past analyses of industry consultation submissions (Neuman *et al*., 2002; Harchard *et al*., 2014; Ulucanlar, 2014; Hiilamo and Glantz, 2015; Lie *et al*., 2018; Ikegwuonu *et al*., 2022; Evans-Reeves *et al.*, 2015), this paper presents analysis of the explicit arguments and implicit tactics used by the tobacco industry in their submissions to the 2022 Scottish Government consultation ‘Vaping Products—Tightening Rules on Advertising and Promoting’, and how these compare to past action by the tobacco industry. As the tobacco industry diversify their product range, it is important to highlight continuations or divergences from past actions and tactics to support efforts to protect the health of the population. This is only the second paper that looks at industry responses to a consultation on e-cigarette policy and the first to examine responses to the 2022 consultation. The first study by Ikegwuonu et al. (Ikegwuonu *et al*., 2022) analysed a range of commercial actors’ engagement with the Scottish Government’s 2014 consultation on e-cigarette regulation’. Our paper, which instead focuses specifically on responses with direct links to the tobacco industry, examines the arguments and tactics used 8 years later in the 2022 Scottish Government consultation. With the market and scale of e-cigarette use expanding rapidly during this period—and given the growing stake of the tobacco industry in the e-cigarette market—it is crucial to examine the evolving tactics and arguments being used by the tobacco industry to influence policy and regulation.

## METHODS

### Data collection

The Scottish Government published all responses where the authors gave permission for publication online on the consultation webpage. All responses answered the same nine questions focusing on the proposed regulations by the Scottish Government. Some of the questions were multi-part, and all were yes or no questions based on one of the government’s proposals followed by an optional free-text section to present reasons for responses.

### Inclusion and exclusion criteria

The inclusion criteria for submissions analysed was based on an organization having direct or indirect links to the tobacco industry as identified via ‘Tobacco Tactics’, the pre-eminent knowledge exchange platform established by the Tobacco Control Research Group at the University of Bath, UK. All submissions were reviewed for these connections and were selected for inclusion in the analysis if they met these criteria by B.H., in discussion with G.H.

Overall, 10 responses were included for the analysis.

### Data analysis

Responses that met the inclusion criteria were downloaded from the Scottish Government consultation webpage and uploaded onto NVivo12.

These responses were subjected to thematic analysis. Thematic analysis allows researchers to compare accounts to identify recurring or common themes in a data set (Gale *et al*., 2013). This approach enables highlighting of important issues, as well as identifying typical responses of particular groups of respondents (Green and Thorogood, 2004), which was a key focus of the research. This analysis was therefore principally ‘bottom up’ with initial codes and themes identified from the data but was also informed by our reading of the previous literature on strategies and tactics used by the tobacco industry to influence government policy (Hatchard *et al.*, 2014; Ulucanlar *et al.*, 2014; Evans-Reeves *et al*., 2015; Hiilamo and Glantz, 2015; Lie *et al*., 2018; Ikegwuonu *et al*., 2022).

After familiarization with the data through reading and re-reading transcripts, initial open coding informed by the previous literature on tobacco industry responses to consultations was conducted by the primary author (B.H.). Secondary coding followed to identify any overlap or inconsistencies and to check there were enough examples in the data set to support these initial codes. Codes were then grouped into themes that reflected patterns and relationships in the data. At each stage, codes and themes were discussed and refined with secondary authors (G.H. and R.P.). Four final overarching themes were identified:

*Theme 1: How industry frames the issue*
*Theme 2: How industry frames the proposals*
*Theme 3: How industry frames its own preferred solutions*
*Theme 4: Implicit overarching tactics used by industry in the submissions*

Subthemes conveyed examples of the tactics used in each of the four categories.

This research therefore analysed the explicit arguments used in the industry submissions as well as the implicit tactics they used to make these arguments.

## RESULTS

The Scottish Government consultation received 757 responses, of which 43 were from organizations. Among these, 24 were from the public/third sector, and 18 were from private organizations, including 4 responses from tobacco companies and 8 additional responses from organizations with direct links to the tobacco industry. Ten of these 12 organizations with tobacco industry links gave permission for their responses to be published. Table 1 shows all the submissions from private organizations and reasons for their inclusion or exclusion.

**Table 1:** : Organization subgroups

Subgroup	Organization name	Reasons for inclusion or exclusion
Tobacco industry	British American Tobacco (BAT) UK	Excluded: BAT did not grant permission for their response to be published in the public domain.
	Imperial Brands Plc (IMB)	Included: Tobacco company
	Japan Tobacco International (JTI)	Included: Tobacco company
	Philip Morris Ltd (PMI)	Included: Tobacco company
Vaping sector	Danish Vapers Association	Excluded: Independent from the tobacco industry
	DripDrop Vapour	Excluded: Independent from the tobacco industry
	LIQUID MIST	Excluded: Independent from the tobacco industry
	Juul Lab	Included: At the time of submission, Altria, the parent company of PMI, owned a 35% share in JUUL and their CEO previously worked at Altria (Tobacco Tactics, 2023b).
	Independent British Vape Trade Association (IBVTA)	Excluded: All IBVTA members are free from any ownership or control by the tobacco and pharmaceutical industries.
	UK Vaping Industry Association	Included: JTI, BAT, PMI, IMB and JUUL Labs are all members of the UKVIA, and their membership makes up a substantial proportion of the UKVIA’s total revenue (Tobacco Tactics, 2022a). A number of directors and senior leaders at the UKVIA have held positions at tobacco companies. UKVIA has also received tobacco industry sponsorship for events (Tobacco Tactics, 2022a).
	VPZ	Excluded: VPZ has close financial links with PMI (Tobacco Tactics, 2023c) but did not give permission for their submission to be published in the public domain.
Other	Association of Convenience Stores (ACS)	Included: The ACS includes BAT, IMB, JTI and PMI as ‘premier club’ members, and JTI has sponsored ACS events (Tobacco Tactics, 2023d).
	Consumer Choice Center (CCC)	Included: A US lobby company that states they are against ‘paternalistic’ government regulations and has received funding from JTI, PMI and BAT (Tobacco Tactics, 2022b).
	NFRN (The Federation of Independent Retailers)	Included: The NFRN do not divulge their members; however, they have received funding from BAT for campaigns against plain packaging and they have collaborated with Imperial on campaigns against illicit trade (Tobacco Tactics., 2020b).
	Pharmacy (unnamed in the consultation document but included as they sell vaping products)	Excluded: Independent from the tobacco industry
	Scottish Grocers Federation (SGF)	Included: The SGF’s membership includes JTI (platinum plus member); BAT, PMU and IMB (gold members); and Juul (bronze member) (Tobacco Tactics., 2023d). In addition, leaked PMI documents from 2012 identified SGF as a ‘media messenger’ in their campaign against plain packaging (Tobacco Tactics., 2023d).
	Scottish Wholesale Association (SWA)	Included: The SWA includes BAT, JTI and IMB as members and has received tobacco industry funding for past campaigns against the government’s plain packaging proposals (Tobacco Tactics., 2020a).
	Winning Scotland	Excluded: Independent from the tobacco industry

The level of detail in the responses differed greatly, with the UKVIA providing the longest submission (8707 words) and the NFRD the shortest (596 words).

Four overarching themes were identified through thematic analysis, each with a number of subthemes (see Table 2).

**Table 2:** : Results

1. How industry frames the issue	2. How industry frames the proposals	3. Actions supported by industry	4. Tactics used by industry in their responses
1.1 Youth vaping is minimal. 1.2 E-cigarettes are a form of harm reduction. 1.3 There is a misperception of harm. 1.4 Illicit trade is the real issue.	2.1 Harm to the public. 2.2 Harm to people from lower socioeconomic backgrounds. 2.3 Poor consequences for the Scottish Government. 2.4 Negative impact on business and innovation.	3.1 Industry should be part of the solution. 3.2 More enforcement of current regulations/ illicit trade. 3.3 Consensus across England and Scotland.	4.1 Proposing delayed action. 4.2 Questioning the evidence base. 4.3 Use of old data. 4.4 Lack of referencing.

### Theme 1: How the industry frames the issues

Overall, the submission responses downplayed the issue of youth vaping, claiming that the numbers were so low that it is not a public concern and also distancing themselves from any responsibility for youth vaping. The responses emphasized the importance of e-cigarettes in reducing the prevalence of smoking in society, framing them as the best cessation tools currently available. They stressed that there is a misperception of harm associated with e-cigarettes, with both the general public and clinicians believing the products are far more harmful than they are in reality. Finally, the responses positioned the real issue as the growth of illicit e-cigarette products and trade.

#### Subtheme 1.1: Youth vaping is minimal and not the fault of industry

As the proposed Scottish Government regulations were primarily in response to concern over the number of children and young people using e-cigarettes, minimizing this worry was a key component of the industry responses.

While the responses from industry stated they strongly supported action that would reduce youth vaping, they countered this by claiming that the number of young people using e-cigarettes regularly was minimal, therefore making the case that further regulation was not necessary.

‘It would be unnecessary to introduce additional measures to prevent against an issue which has not arisen significantly in the market. Youth access remains negligible, and it is essential that enforcement bodies are adequately equipped to enforce existing regulations, before increasing their burden in areas which are more tangential to the issues of youth access.’ [UK Vaping Industry Association (UKVIA)]

There was a tendency in the submissions to use youth vaping statistics from a few years ago. These were not a good representation of the current situation, given that a substantial increase in e-cigarette use had occurred in the last couple of years, and more up-to-date data were available at the time of submission.

‘Looking across to the SALSUS [Scottish Schools Adolescent Lifestyle and Substance Use Survey] 2018, we note that regular use of e-cigarettes among 13 and 15 years olds was found to be very low at 2% and 3% respectively.’ [Philip Morris Ltd (PMI)]

The responses from industry consistently argued their actions were not to blame for any underage e-cigarette use, therefore disputing the need for further regulation:

‘We do not market our vapour products to youth or non-smokers / non-vapers, and we do not market or design e-liquids in flavours that appeal primarily to youth.’ (Imperial)

#### Subtheme 1.2: E-cigarettes as a form of harm reduction

The industry responses consistently position e-cigarettes as a smoking cessation tool and a form of harm reduction, stating that increased regulations could negatively impact this. The responses referred to users of e-cigarettes as current or former smokers who would benefit from using these products instead of cigarettes. They largely ignored that young people and non-smokers also use their products and would not benefit from any harm reduction potential.

The Consumer Choice Centre referred to e-cigarettes as a ‘life-saving alternative’, and the Scottish Wholesale Association (SWA) said that there is ‘a clear health imperative to sell these legal products as an effective cessation device’, warning against ‘restricting the biggest influencer in helping smokers to quit smoking’.

#### Subtheme 1.3: There is a misperception of harm associated with e-cigarettes

Industry responses often referred to a misperception of harm surrounding e-cigarettes. While the current evidence base appears to show that e-cigarettes are far less harmful than cigarettes, the industry responses state that this is not understood by the public or clinicians who greatly overestimate the risk of product use.

The responses regularly blame the media for reporting ‘misinformation’ with the UKVIA response claiming that ‘clinicians are relatively unaware as to the relative harm of e-cigarettes’, thus stopping them from reaching their potential to support smoking cessation. The responses argue that increased regulation will ‘exacerbate existing levels of misinformation about vaping and other less harmful alternatives to smoking, thereby discouraging those who would otherwise continue to smoke from switching’ (PMI).

Industry responses also highlighted differing levels of misperception in society, suggesting that benefit from a less harmful alternative to smoking to those from lower socioeconomic backgrounds could be reduced.

‘Among poorer groups there is more widespread misperceptions about the relative harm of smoking versus vaping.’ (Juul)

#### Subtheme 1.4: Illicit trade is the real issue

The responses from industry regularly argue that the dangers associated with use of e-cigarettes and products that appeal to young people are due to the illicit market and unregulated e-cigarette products. These submissions additionally claim that unregulated e-cigarette products are putting users in danger and that criminal enterprises are benefitting from their sales. ‘Illicit trade and unregulated products are blamed for ‘common misperceptions preventing those adult smokers considering making the switch to a less harmful alternative’ [Japan Tobacco International (JTI)].

‘Instead of further regulation of legal e-cigarette companies, the submissions ask that government focus on tackling those bad actors in the industry which currently disregard existing regulation’ (UKVIA). The responses argued that further regulation on already regulated e-cigarette companies would play into the hands of the illicit market as it ‘could push the market underground and make things worse’ (SWA).

### Theme 2: How the industry frames proposals

The industry responses were unsurprisingly very critical of the Scottish Government’s proposals. Despite the government’s aim to reduce the uptake of e-cigarette use in non-smokers and young people, the industry argued that these proposals would lead to harmful consequences for the public, in particular those of lower socioeconomic status, as well as for the government and industry.

#### Subtheme 2.1: Harm to the public

The industry responses argue that the proposals would reduce smokers’ access to and awareness of e-cigarettes and therefore their ability to benefit from a less harmful alternative.

‘IMB does not support proposals which we believe will severely impact the ability of existing adult smokers from transitioning to a potentially reduced risk product, by limiting their access to responsible, regulated information on the vape category.’ (Imperial)

The submissions argue the proposals will lead to fewer smokers transitioning to e-cigarettes and will therefore have the opposite outcomes to what the government intended.

‘Removing or restricting the ability for smokers to see or purchase a vape product could have the adverse effect and unintended consequence of pushing people back to smoking.’ (SWA)

#### Subtheme 2.2: Harm to people from lower socioeconomic backgrounds

Some responses argued that the proposals would disproportionately affect the most disadvantaged communities as they have a higher prevalence of smoking tobacco and therefore the highest need for smoking cessation support. These responses stated that the regulations would increase the existing large gap in knowledge of the less harmful alternatives available and will therefore perpetuate health inequalities. In addition to the knowledge gap, the responses argued that Scottish proposals to ban free samples would economically impact this group.

‘Free distribution and nominal pricing are exceptionally important for smokers from lower socio-economic groups, where affordability of less harmful alternatives is important.’ (UKVIA)

#### Subtheme 2.3: Negative consequences for the Scottish Government

Many of the responses referenced Scotland’s smokefree generation target and that Scotland is unlikely to reach their 2034 target. Submissions claim that the regulations will reduce the amount of smokers who make the switch and will therefore further reduce progress towards the smokefree goal.

Some of the responses also referred to the ‘burden’ of the proposals for the government, in particular in enforcement. They argued trading standards were underequipped and underfunded to deal with additional requirements.

‘Compliance would be an issue for current enforcement bodies within existing regulations, and ensuring the burden on these bodies does not increase will go further towards mitigating the perceived issues raised in the consultation.’ (UKVIA)

#### Subtheme 2.4: Negative impact on business and innovation

The impact on business was less frequently discussed in the responses than the impact on the public and government. When the negative impacts were mentioned, it tended to be framed as having a subsequent negative impact on society; for example, restrictions ‘would limit the agility of the industry and preclude further product development to the detriment of smokers looking to quit’ (UKVIA).

Some responses also argued against the ‘overmedicalization’ of e-cigarettes from the proposals, stating this would put smokers off the products. Instead, they maintained that ‘for the benefits of e-cigarettes as a smoking cessation method to be realised, they must be able to operate in a properly functioning competitive market’ (SWA).

### Theme 3: Actions supported by industry

There were a number of preferred courses of action proposed in the responses from industry in place of the Scottish Government’s proposals, which are discussed in the following sections.

#### Subtheme 3.1: Industry should be part of the solution

Industry frequently positioned themselves as smoking cessation professionals whose expertise should inform policy. The UKVIA claimed that the proposed regulations ‘misunderstand the business models of vape retailers who provide stop smoking support advice and services’.

They stated the public preferred accessing e-cigarettes in a retail setting than a pharmacy and in addition, this would ease pressure on the NHS. There was also the suggestion that there was much confusion about e-cigarettes among the public and clinicians and therefore industry was needed to correct these misperceptions.

Additionally, the responses regularly referred to industry-developed guidance and schemes such as ‘Challenge 25’ for dealing with the problem of youth vaping. Many of the responses also referred to industry’s own guidance that has been developed to reduce youth vaping, but it was not clear what the guidance entailed or how it was being implemented or monitored.

‘UKVIA has produced guidance on underage sales for vape retailers and marketing guidelines, which provide effective guidance to all responsible retailers to ensure vape products remain visible to those adult smokers looking to quit or transition without appealing to youth and non-smokers or vapers’. (UKVIA)

#### Subtheme 3.2: More enforcement of current regulation/illicit trade

The responses often argued that the current e-cigarette regulations were sufficient, claiming that there was no need for additional regulations: ‘the current regulatory framework on vape products, supplemented by the additional guidance available to the industry, is sufficient in ensuring youth access prevention’ (Imperial).

The responses argued that instead of introducing new regulations, it would be better to enforce the current ones, which they claimed were largely not being enforced. The focus on enforcement by the industry referred mainly to illicit trade and unregulated products.

‘IMB suggests to the Scottish government that further provisions are made to enable Trading Standards to effectively enforce current regulations, rather than implementing further restrictive regulations to be enforced’. (Imperial)

#### Subtheme 3.3: Consensus across England and Scotland

The responses made numerous comparisons between the action being taken in Scotland and that in England. Many industry responses were supportive of England, which was at an earlier stage than Scotland in terms of regulation, stating that their actions were progressive. Some of the responses stated that England made better use of the evidence and was more aware of the role of e-cigarettes as smoking cessation tools and as a reduced risk alternative.

Despite health being a devolved power, some of the submissions called for a unified approach across the UK on e-cigarette regulation.

‘It is important to avoid establishing an entirely separate regulatory regime in Scotland, as this will undermine the strength of the provisions in the TPD (Tobacco Products Directive) which are already exceptionally prescriptive. A separate regulatory environment will also contribute to existing misinformation and will undermine the potential of e-cigarettes as an effective tool to stop smoking.’ (UKVIA)

### Theme 4: Tactics used by industry in their responses

#### Subtheme 4.1: Proposing delayed action

The responses regularly asked the government to commission more evidence or research to justify their regulations. This implicitly suggested a delay in regulation being implemented or even attempted to deter government action entirely as high-quality evidence is often timely and costly.

‘ACS recommends that the Scottish Government commissions a study that establishes where youths are accessing e-cigarettes from’. [Association of Convenience Stores (ACS)]

‘We would recommend the Scottish Government undertakes new detailed research regarding public attitudes to vape devices and advertising restrictions, such to better appreciate the public perceptions and attitudes which will influence policy decisions.’ (UKVIA)

#### Subtheme 4.2: Questioning the evidence base

The industry responses took issue with some of the evidence used in the government consultation document and how some of the statistics were presented, for example, arguing that relative risk should be the starting point to show e-cigarette harm reduction potential in comparison to smoking tobacco. Evidence included in the consultation report from the WHO (WHO, 2020) is singled out in a few responses as not aligning with the UK evidence base.

‘Some statements included in the consultation document do not reflect this generally accepted view accurately and might go some way to confusing or deterring existing smokers from deciding to transition away from combustible tobacco.’ (Imperial)

#### Subtheme 4.3: Use of old data

As outlined above, the industry submissions often did not use the most recently available data on e-cigarette use in young people. Given the rise in use was very recent, data even a couple of years old did not show the true extent of the issue.

#### Subtheme 4.4: Lack of referencing

None of the submissions bar one included a list of references. It was therefore difficult to check the accuracy of industry claims, the validity of the research mentioned or the existence of conflicts of interest within these studies.

## DISCUSSION

### Summary of findings and comparison to historical industry tactics

Thematic analysis of industry responses to the Scottish Government’s consultation found a range of arguments, framings and tactics used to attempt to influence the proposed increased regulations on e-cigarettes. A number of subthemes sat under the four overarching themes: (1) *how industry framed the issue*, (2) *how they framed the proposals*, (3) *actions supported by industry* and (4) tactics used by industry in their responses. Overall, very similar arguments, framing and tactics were used across all of the industry responses, suggesting some level of cooperation may have taken place.

The industry submissions were unanimous in stating that they were against young people using e-cigarettes. However, they questioned whether youth vaping was a real concern by arguing that the number of young people doing so was so low, often selecting to use figures that were more favourable to their arguments. ASH data showing a year-on-year increase in the number of young people vaping (ASH, 2023b) (published each year) was omitted from any of the submissions.

There is substantial research demonstrating the tobacco industry’s use of industry-funded studies (Evans-Reeves *et al.*, 2020; Hatchard *et al*., 2014). This tactic has received a lot of attention and criticism from researchers in the past and therefore it appears that industry is seeking to use more credible data sources to avoid this criticism. The submissions tended to reference credible surveys, such as SALSUS, to highlight the prevalence of youth vaping, although no submission included full reference lists and often this was not the latest figures available at time of submission. By opting to use figures that were years old or by using figures associated with a younger age group (e.g. 13- to 15-year olds), they were able to mask the recent large rise in use, as well as much greater use in 16- to 17-year olds. There were many other instances when the submissions referred to research or clinical studies without giving a reference, meaning that it was not possible to check for accuracy or conflicts of interest. Denying the scale of use appeared to be a new tactic and has not been explored in past research, so it will be important that public health practitioners and policymakers are up to date with the latest figures and rates of increase in e-cigarette use, particularly among young people.

The industry responses argue that they are not attempting to attract youth users and therefore are not to blame for the increase in youth vaping. In their submission, the tobacco company Imperial claim that they do not market products or flavours to appeal to young people or non-smokers, for instance. However, Blueberry Sour Razz and Watermelon Ice are among their current product range, and the ASH survey showed that fruit flavours are the most popular for young people, 60% of whom favoured them (in comparison to 17% for the second most popular option of sweets/candy flavour) (ASH, 2023b); young people’s preference for fruit flavours has been shown in studies elsewhere (Gendall and Hoek, 2021; Notley *et al*., 2021). Many of the submissions incorrectly state young people favour flavours such as candy floss rather than the fruit flavours that make up the majority of their product range. In their submission, JTI state that ‘activities should ensure e-cigarettes do not appeal to under 18s, which could be conveyed through the use of comic or cartoon characters, toys, or sweets for instance’. This is arguably a simplistic depiction of what is appealing to children and young people and ignores the fact that many young people can be attracted to products that can make them feel grown up.

It has been a long-standing tactic used by the tobacco industry to outwardly state that young people should not smoke and that it is an ‘informed adult choice’ (Hoek *et al*., 2020). This of course ignores that addiction is not a choice and that association with adult choices is part of the appeal to young people (Ling and Glantz, 2002). The youth market is very important to the tobacco industry as young people are potential lifelong customers, and the industry recognizes this period in life as crucial in the solidification of their status as a smoker (Perry, 1999). As a 1981 PMI internal document stated: ‘Today’s teenager is tomorrow’s potential regular customer’ (Henningfield *et al*., 2006). Truth Tobacco Industry Documents demonstrate that the industry views smoking initiation as beginning in adolescence, and they therefore historically have used strategies to appeal to young people, including developing ‘starter brands’ linked to youth and rebellion (Ling and Glantz, 2002). As smoking is decreasing in high-income countries, particularly among young people, the tobacco companies are arguably trying to guarantee their future by promoting vaping to new generations. This includes use of marketing and flavours that appeal primarily to young people.

An area of complete omission across the consultations was any discussion of addiction to e-cigarettes in young people. The ASH survey reported that 8.6% of young people who currently vape have extremely strong urges to do so in comparison to 3.4% of young people who smoke cigarettes (ASH, 2023b). Public health professionals and policymakers need to pay attention to the financial, social and educational impacts that addiction to vaping is having on young people.

As highlighted in Ikegwuonu *et al*.’s study (Ikegwuonu *et al*., 2022), research industry submissions continue to position e-cigarettes as a harm reduction smoking cessation tool that can support the health outcomes of the population. The majority of the submissions stated that their target group is solely current or ex-smokers. They do not refer to the fact that over 8% of regular e-cigarette users are never smokers or that a growing number of young people are using their products (ASH, 2023a,b). The submissions also regularly promote dual use of e-cigarettes with tobacco, which contradicts their harm reduction framing as the evidence shows that dual use is not a good method to quit smoking or protect health (Jackson *et al*., 2020).

A harm reduction argument may be convincing for policymakers but masks the fact that, for the tobacco industry, the main priority ‘is the maximisation of sales, profit and return to shareholders, which places them at odds with serving a mandate of harm reduction’ (Dewhirst, 2021). Critics argue that the tobacco industry’s entry into the e-cigarette market is a market expansion strategy to diversify their products rather than a genuine harm reduction strategy (Dewhirst, 2021). Research shows that the tobacco industry’s historic adoption and use of the term ‘harm reduction’ occurred in response to the public health agenda (Peeters and Gilmore, 2015) and allowed the industry to participate in dialogue with scientists and policymakers by presenting itself as a partner (Peeters and Gilmore, 2015). In addition, it was used as a corporate social responsibility strategy to improve their tarnished reputation and position themselves as responsible businesses (Peeters and Gilmore, 2015). This mirrors what the industry is attempting to achieve in these consultation submissions; by positioning themselves as experts in harm reduction and smoking cessation, they want to ensure that they are a part of the solution and to portray themselves as responsible actors.

The tobacco industry has previously promoted ‘less harmful’ products such as ‘low-tar’ cigarettes. Created to encourage concerned smokers to switch brands rather than quit, they have been found to be as harmful as standard brands (Yang, 2014). Influenced by industry-sponsored research and harm reduction marketing, many people, particularly in China, continue to smoke these brands due to the perception of it being a safer alternative (Yang, 2014). While research largely suggests that e-cigarettes are much less harmful than cigarettes, long-term effects are still unknown, and addiction and dependence to new products can bring about new harms. The tobacco industry’s supposed commitment to harm reduction can also be questioned by the fact they continue to aggressively market cigarettes in low- and middle-income countries, where rates of smoking continue to rise without the stringent tobacco control that exists in high-income countries (ASH, 2019).

The submissions argue that increased regulations on legal e-cigarettes will play into the hands of illicit trade. This is a spurious argument that the tobacco industry used frequently during the SP debate (Evans-Reeves *et al.*, 2020; Ulucanlar *et al*., 2014; Lie *et al*., 2018). In submissions to the government’s SP consultation, the tobacco industry claimed SP would lead to an increase in illicit trade and unregulated cigarettes in the market; these submissions relied upon industry-funded research, and the extent of illicit trade was greatly overestimated (Evans-Reeves *et al.*, 2020; Lie *et al*., 2018). Studies have also shown that tobacco companies managed to infiltrate UK newspaper coverage to suggest that the illicit tobacco trade was booming during the SP debates in order to bring about opposition to the SP proposals (Evans-Reeves *et al*., 2020).

In the submissions analysed here, industry advocated for a UK-wide strategy to e-cigarette regulation rather than separate strategies in Scotland and England. This could be seen as a delaying tactic, as England is behind Scotland in regulating e-cigarettes. A similar argument was identified in Holden and Hawkins’s research (Holden and Hawkins, 2013) on the alcohol industry, devolution and minimum unit pricing (MUP) in Scotland. They show that industry’s opposition to Scotland adopting MUP was presented as a concern of efficiency and uniformity; however, in reality, it was ‘an attempt to shift the location of the debate from Scotland – where there was sympathy among the government for price-based interventions and an active PH [public health] lobby supporting the policy – to Westminster where there was less vocal support and a Conservative-led government sceptical about the benefits of government intervention in the market’ (Holden and Hawkins, 2013, p. 255). Scotland was also a pioneer in implementing smokefree legislation in public spaces before it spread to the rest of the UK (ASH, 2022), so the fear of policy spread is likely to be of concern to the tobacco industry in the case of e-cigarette regulation.

Many of the industry submissions advocate for their participation in the development of regulations either through partnerships with the policymakers or through the adoption of industry-developed guidance as seen in Ikegwuonu *et al*.’s study (Ikegwuonu *et al*., 2022). For example, Imperial states ‘we would advocate inclusion of industry representatives in a steering group or “enforcement board” in order to ensure the seamless flow of relevant information between industry and enforcement authorities’. There is also frequent promotion of the industry-developed ‘Challenge 25’ guidance as the solution to youth vaping. Leaked PMI documents from 2014 on its long-term strategy state that in an attempt to improve its image, the organization would position itself as a ‘trusted and indispensable partner, leading its sector and bringing solutions to the table’ (Hird *et al*., 2022). For instance, the tobacco industry positioned itself as part of the solution in dealing with illicit cigarette trade, which it later emerged that they were a part of (Hird *et al*., 2022). Offering expertise and advice and partnerships with government can be seen as an attempt to override Article 5.3, allowing the industry a means to exert hidden powers and influence policy in a way that will be financially and reputationally beneficial to them (Hird *et al*., 2022). The Department of Health and Social Care (DHSC) has very recently updated its guidance on how to limit interactions with the tobacco industry, which includes organizations or individuals with commercial or vested interests in the industry (DHSC, 2023). However, the guidance on consultations is extremely minimal stating only that ‘When undertaking a consultation on tobacco policy, respondents should be asked to declare any direct or indirect links to, or funding received from, the tobacco industry’ (DHSC, 2023). As discussed above, this still gives the tobacco industry a great deal of freedom in how they present evidence and arguments.

### Implications and recommendations for policy makers and researchers

#### Policymakers

- As called for by other researchers (Hatchard *et al*., 2014; Evans-Reeves *et al*., 2015; Ikegwuonu *et al*., 2022), this study suggests that better protection is needed for public health consultations from the vested interests of industry. Responding to consultations appears to be a loophole in the FCTC’s Article 5.3, but the major conflicts of interest involved—as well as historic actions of the tobacco industry—should prevent this from being allowed.
- In the meantime, there are actions that could be taken immediately to regulate how industry can respond to government consultations. This should include making referencing mandatory to allow policymakers to fact-check and highlight when industry-funded research is used.
- The DHSC guidelines on interactions with the tobacco industry are very sparse in their section on consultations. This should be expanded, informed by public health research on the commercial determinants of health.
- Some of the tactics used in industry submissions may be missed by busy policymakers working on tight deadlines. Therefore, additional training to improve awareness of these tactics or closer working with researchers may help to reduce scope for the tobacco industry to influence policy making.

#### Researchers

- As British American Tobacco (BAT) is such a large player in the e-cigarette market, it would be useful to know what arguments and tactics were used in their submission. Therefore, future research could consider using methods to obtain withheld submissions such as Freedom of Information requests.

### Limitations

This study could only examine responses that had permission to be published on the Scottish Government website, and, as a result, responses from BAT and VPZ were omitted as they did not give permission for publication. In 2021, BAT owned 17.4% of the global cigarette market, which is particularly large when compared to PMI’s 0.2%, JTI’s 1.5% and Imperial’s’s 2.8% (Tobacco Tactics, 2023a). With such large stakes in the market, it would have been particularly interesting to see their submission, raising questions about why the organization chose not to have it in the public domain. As shown in the literature though, tobacco companies tend to use very similar arguments and tactics, so it is likely the other responses analysed are a good indicator of what may have been included in the BAT submission. A potential limitation in the analysis stage was not doing initial double coding; however, as mentioned above, themes were discussed and refined with secondary authors.

## CONCLUSION

Building on the extensive body of research on tobacco industry practices, this research focuses on the industry’s attempts to influence e-cigarette policy in Scotland. It is the first study examining the 2022 Scottish Government's consultation on E-cigarette regulation, highlighting the explicit arguments and implicit tactics used by the tobacco industry and linked organizations in their consultation submissions. It is important that policymakers and the public are aware of these, so that they can restrict the industry’s undue influence over the public health of society.

E-cigarette use is still a relatively new phenomenon, and currently little is still known about how the tobacco industry engages with and attempts to influence its specific regulation. As rates of e-cigarette use continue to increase though, and with the tobacco industry’s share in this market increasing, it is essential to understand how the tobacco industry is continuing with historic arguments and tactics and whether new strategies have emerged.

This analysis of industry responses to the Scottish Government’s consultation showed that overall there was a continuation of well-documented tactics and arguments historically used by the tobacco industry. This is despite the fact that the industry argued they were promoting harm reduction and smoking cessation in society, most likely in an attempt to improve their reputations and influence while increasing profit through product diversification and potential new customers. However, our study has also highlighted a number of divergences or new tactics employed by industry not previously reported in the tobacco control literature, including use of old data to downplay the issues and attempts to encourage UK-wide policy, thus delaying Scotland from taking pre-emptive action. Increased awareness of—and restrictions on—the tobacco industry’s ability to respond to government consultations is crucial to protect public health from vested commercial interests.

## Data Availability

All data analysed in this project are available in the public domain.
